# Procatechuic acid and protocatechuic aldehyde increase survival of *Caenorhabditis elegans* after fungal infection and inhibit fungal virulence

**DOI:** 10.3389/fphar.2024.1396733

**Published:** 2024-05-22

**Authors:** Chunyan Yuan, Yuxing Wang, Le Zhang, Dayong Wang

**Affiliations:** ^1^ Department of Gynaecology and Obstetrics, Zhongda Hospital, Southeast University, Nanjing, China; ^2^ Deaprtment of Biochemistry and Molecrla Biology, School of Medicine, Southeast University, Nanjing, China

**Keywords:** *C. elegans*, procatechuic acid, protocatechuic aldehyde, *C. albicans infection*, virulence

## Abstract

Protocatechuic acid (PCA) and protocatechuic aldehyde (PAL) are important phenolic compounds in plants. We here investigated their possible beneficial effect against fungal infection and the underlying mechanism. The model animal of *Caenorhabditis elegans* was used as host, and *Candida albicans* was used as fungal pathogen. The nematodes were first infected with *C. albicans*, and the PCA and PAL treatment were then performed. Post-treatment with 10–100 μM PCA and PAL suppressed toxicity of *C*. *albicans* infection in reducing lifespan. Accompanied with this beneficial effect, treatment with 10–100 μM PCA and PAL inhibited *C. albicans* accumulation in intestinal lumen. In addition, treatment with 10–100 μM PCA and PAL suppressed the increase in expressions of antimicrobial genes caused by *C. albicans* infection. The beneficial effect of PCA and PAL against *C. albicans* infection depended on p38 MAPK and insulin signals. Moreover, although treatment with 10–100 μM PCA and PAL could not exhibit noticeable antifungal activity, PCA and PAL treatment obviously suppressed biofilm formation, inhibited hyphal growth, and reduced expressions of virulence genes (*ALS3*, *CaVps34*, *Vma7*, *Vac1*, and/or *HWP1*) related to biofilm formation and hyphal growth in *C. albicans*. Therefore, our data demonstrated the potential of PCA and PAL post-treatment against fungal infection and fungal virulence.

## Introduction

Based on clinical survey, in the United States, *Candida* spp are considered as the fourth most common cause for systemic infections with high mortality in hospital (Pfaller and Diekema, 2010). *Candida albicans*, a fungal pathogen, can be widely detected in human microbiome (Mayer et al., 2013). In clinical, *C. albicans* can result in some forms of infections, including the life-threatening systemic infection (Nobile and Johnson, 2015; Lopes and Lionakis, 2022). Some virulence factors, such as biofilm formation and hyphal growth, contribute to pathogenic potential of *C. albicans* (Gow et al., 2003; Pereira et al., 2021). Thus, how to reduce and counteract *C. albicans* infection is an important issue in the clinical.

The model animal of *Caenorhabditis elegans* relies on innate immunity mechanism to defend pathogen infection (Martineau et al., 2021). It can provide a useful platform for determining interactions between hosts and bacterial or fungal pathogens (Kumar et al., 2020). Antimicrobial proteins secreted by different tissues act as immune effectors in nematodes after pathogen infection (Millet and Ewbank, 2004; Dierking et al., 2016). In nematodes, several signaling pathways (such as p38 MAPK and insulin) have been identified to regulate the innate immunity (Kim and Ewbank, 2018). It has been suggested that *C. elegans* can be further used for the study of human infectious diseases (Marsh and May, 2012).

The model animal of *C. elegans* is an important model for pharmacological discovery for some diseases (Griffin et al., 2017; Bulteriis and Braeckman, 2020). Due to short lifespan and life-cycle and exposure to a small amount of compound, it can be used for large-scale or high-throughput pharmacological and toxicological screens (O’Reilly et al., 2014; Carretero et al., 2017; Wang, 2020). Meanwhile, *C. elegans* has been widely applied for screening and identifying novel agents or compounds with the functions to enhance host immune response and to attenuate microbial virulence (Arvanitis et al., 2013).

After fungal infection, lifespan of nematodes could be reduced (Kim et al., 2020). Infection with *C. albicans* can induce antifungal immune defenses by activating expression of some antimicrobial genes (Sun et al., 2016b). Mutation of *pmk-1* and *daf-16* caused susceptibility to fungal infection (Pukkia-Worley et al., 2011; Kitisin et al., 2022), suggesting that PMK-1/p38 MAPK and DAF-16 in insulin signaling pathway mediate the resistance to *C. albicans* infection. For *C. elegans*, it can also be used for pharmacological assessment of compound against fungal infection, including *C. albicans* infection (Anastassopoulou et al., 2011; Madende et al., 2020).

Protocatechuic acid (PCA) and protocatechuic aldehyde (PAL) are two phenolic compounds. PCA and PLA can be found in herbs, fruits, and vegetables (Kakkar and Bais, 2014). Some aspects of beneficial effects of PCA and PAL, including antioxidation and anti-inflammation, have already been suggested (Zhang et al., 2021; Li et al., 2022). However, the possible usefulness of PCA and PLA treatment against fungal infection and underlying molecular basis remain largely unclear. Thus, we aimed to further determine possible beneficial effect of PCA and PAL against fungal infection in hosts and underlying mechanism. In this study, *C. albicans* was used as the fungal pathogen, and *C. elegans* was employed as the host.

## Materials and methods

### Maintenance of nematodes

Nematodes (wild-type N2) were cultured normally on nematode growth medium (NGM) plates seeded by *Escherichia coli* OP50 (Brenner, 1974). *C. elegans* strain was purchased from *Caenorhabditis* Genetics Center (CGC). The NGM plates were prepared as described (Stiernagle, 2006). To prepare synchronized young adults for fungal infection and following pharmacological treatment, the gravid nematodes were lysed with lysis buffer (2% HOCl and 0.45 M NaOH) to obtain the embryos (Zhao et al., 2022a). Collected embryos were transferred onto new NGM plate to allow to develop into young adults.

### Fungal preparation

Information for *C. albicans* strains was shown in Supplementary Table S1. If not specially indicated, the used *C. albicans* strain is SC5314, which has been shown to be virulent for nematodes (Sun et al., 2015). Fungal strains were cultured in liquid yeast extract-peptone-dextrose broth or on brain heart infusion agar containing kanamycin (45 mg/mL).

### Fungal infection

Young adults were transferred on BHI agar plates containing kanamycin (45 mg/mL) and seeded with *C. albicans*. PBS buffer (200 μL) was added together with 50 μL SC5314 to facilitate fungal dispersion. *C. albicans* infection was performed from young adults for 48-h at 20°C.

### Pharmacological treatment

The PCA and PAL (purity, ≥98%) were purchased from Weikeqi Bio-Technology Co., Ltd. (China). After *C. albicans* infection, animals were treated with PCA and PAL for 24-h at 20°C. After pharmacological treatments, animals were cultured on NGM plate. Used concentrations for PCA and PAL were 10, 50, and 100 μM as described (Kong et al., 2014; Han et al., 2019).

### Assay of lifespan

Lifespan of *C. elegans* was analyzed as described (Wang et al., 2023). After PCA or PAL treatment, survival of animals was checked every day. Animals were considered as dead if no responses were observed after prodding using a platinum wire. Median lifespans refer to days at which 50% nematodes survive. Fifty nematodes were tested for lifespan assay. Three replicates were performed.

### Colony-forming unit (CFU) assay

*C. albicans* CFU was quantified in nematodes as described (Sun et al., 2016a). After infection and PCA and PAL treatments, animals were washed for five times using M9 buffer to remove fungal lawn on surface. Each group of fifty animals was homogenized and transferred on a YPD agar containing kanamycin (45 μg/mL), ampicillin (100 μg/mL), and streptomycin (100 μg/mL). After incubation for 48-h at 37°C, numbers of fungal colony were counted. Ten replicates were carried out.

SC5314:GFP accumulation in animal’s body was also examined. Data was expressed as relative fluorescence intensity of SC5314:GFP in intestinal lumen, which was normalized to autofluorescence of intestine. Forty animals were tested for each group. Three replicates were performed.

### Transcriptional expression analysis

Using RNeasy Mini Kit (Qiagen), total RNAs of *C. elegans* and *C. albicans* were extracted for cDNA synthesis. To isolate biofilm cell RNA, the yeast cell suspensions (1 × 10^6^ CFU/mL) were incubated with fresh RPMI 1640 in 96-well plates for 3-h at 37°C. Supernatants were then removed, and wells were washed using PBS buffer to remove unattached cells. PCA or PAL was added into wells and incubated at 37°C for 24-h. The 430–600 μm glass beads were applied to break adherent cells. To isolate hyphae cell RNA, the suspensions (1 × 10^6^ CFU/mL) were collected and incubated with PCA and PAL diluted with RPMI 1640 containing 10% FBS at 37°C for 10-h. *C. albicans* cells were collected by centrifugation (3000 rpm, 2-min). Quality of RNAs was assessed by the ratio of OD260/280 in Nanodrop One. SYBR Green master mix was used for quantitative real-time polymerase chain reaction (qRT-PCR). Comparative cycle threshold method was employed. Internal reference gene (*tba-1*) expression was normalized in nematodes (Zhao et al., 2022b). Gene encoding 18S rRNA was used as internal reference gene in *C. albicans* (Shin and Eom, 2019). Expression of genes in control group was normalized to 100%. Primer information was shown in Supplementary Table S2, S3. Three replicates were performed.

### RNA interference (RNAi)

RNAi was performed by feeding animals with *E. coli* HT115 expressing *daf-16* or *pmk-1*. RNAi was carried out after the SC5314 infection. *E. coli* HT115 expressing L4440 (empty vector) acted as the control (Xu et al., 2022). Efficiency of RNAi was assessed by qRT-PCR (Supplementary Figure S1).

### Antifungal activity of PCA and PAL

(1) Time-kill assay. Method was performed as described (Sun et al., 2016a). *C. albicans* SC5314 cells cultured overnight were suspended in RPMI medium to reach the concentration of 1–5 × 10^4^ cells/mL. PCA and PAL were added to inoculated RPMI medium to obtain anticipated concentrations. Again, the centrifugated SC5314 cells were dispensed into culture tubes containing PCA and PAL in a volume of 5 mL. The *C. albicans* cells were incubated at 35°C. After PCA or PAL treatment, colony counts of SC5314 were analyzed on YPD agar at 6, 12, 18, and 24-h. Fluconazole was employed as the control. Experiments were carried out in triplicate.
(2) Agar diffusion assay. Method was performed as described (Lafleur et al., 2013). After concentration by centrifugation, 10^7^ cells/mL for *C. albicans* SC5314 were inoculated in liquid YPD medium. The 10 mL suspension was transferred on YPD agar plates. PCA or PAL (50 μL) was pipetted on filter disks (6-mm diameter) and placed onto agar surfaces. Plates were incubated for 48-h at 35°C. Fluconazole (50 μL) was employed as the control. Experiments were performed in triplicate.

### Fungal biofilm formation

After PCA and PAL treatment, *C. albicans* biofilms in 96-well plate were first washed by PBS buffer. The biofilms were fixed by methanol (200 μL), and stained by crystal violet (0.1%) for 5-min. After staining, each well in the plate was washed with sterile distilled water for three times. The wells were dried for 1-h at 60°C, and the biofilm was dissolved by acetic acid (33%). To further quantify formation of biofilm, absorbances at wavelength of OD_600_ were measured. Experiments were repeated three times. Biofilm formation was also visualized under the light microscope.

### Fungal hyphal growth

The hyphal growth assay was analyzed as described (Manoharan et al., 2017). After incubation of yeast cell suspension (1 × 10^6^ CFU/mL) with PCA or PAL for 10-h with agitation (200 r/min) at 37°C, *C. albicans* hyphae growth was visualized under a light microscope. RPMI 1640 containing 10% FBS was employed as the control. Experiments were repeated three times.

### Data analysis

Statistical analysis was performed by SPSS 12.0 software. Difference between different groups was examined using analysis of variance (ANOVA). Probability level of 0.01 was considered statistically significant. Statistical significances between lifespan curves were analyzed by Kaplan-Meier survival analysis, followed by log-rank test.

## Results

### Role of PCA and PAL in promoting lifespan in nematodes after fungal infection

**FIGURE 1:**
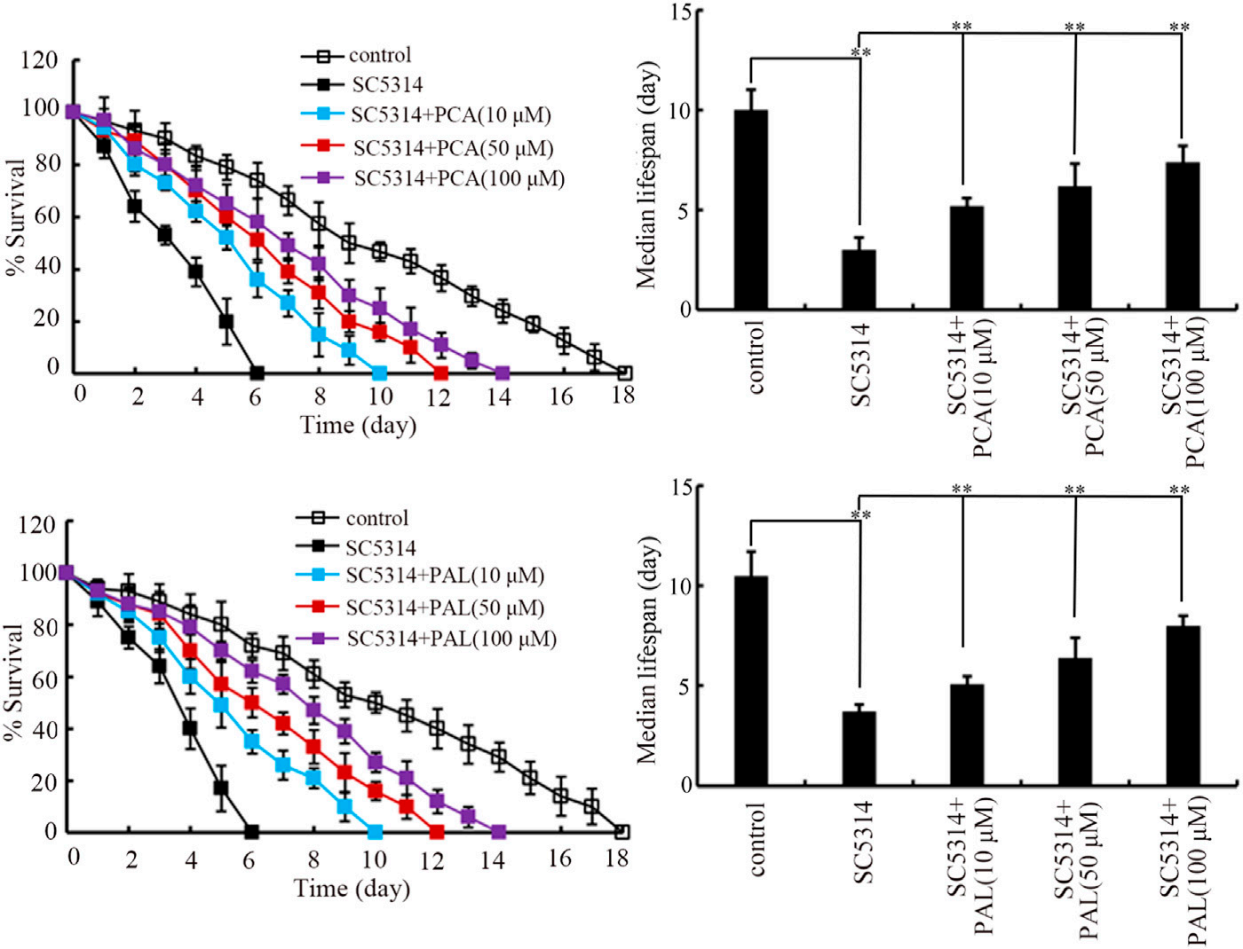
Effect of PCA and PAL treatment on lifespan of nematodes after *C. albicans* infection. PCA, protocatechuic acid; PAL, protocatechuic aldehyde. ^**^ *p <* 0.01. Lifespan curves of SC4314 showed a significant difference (*p* < 0.01) compared to control. Lifespan curves of SC5314+PCA (10 μM), SC5314+PCA (50 μM), SC5314+PCA (100 μM), SC5314+PAL (10 μM), SC5314+PAL (50 μM), and SC5314+PAL (100 μM) showed a significant difference (*p* < 0.01) compared to SC5314.

After SC5314 infection only, the lifespan was sharply decreased by SC5314 from day-2 (Figure 1). The lifespan reduction observed in SC5314 infected nematodes could be significantly suppressed by treatment with PCA and PAL at concentrations of 10–100 μM (Figure 1). In addition, the effect of PCA and PAL to extend lifespan of SC5314 infected nematodes was concentration dependent (Figure 1). This observation demonstrated the effect of PCA and PAL treatment against toxicity of fungal infection in decreasing lifespan.

### Role of PCA and PAL against *C. albicans* colony formation in *C. elegans*

**FIGURE 2:**
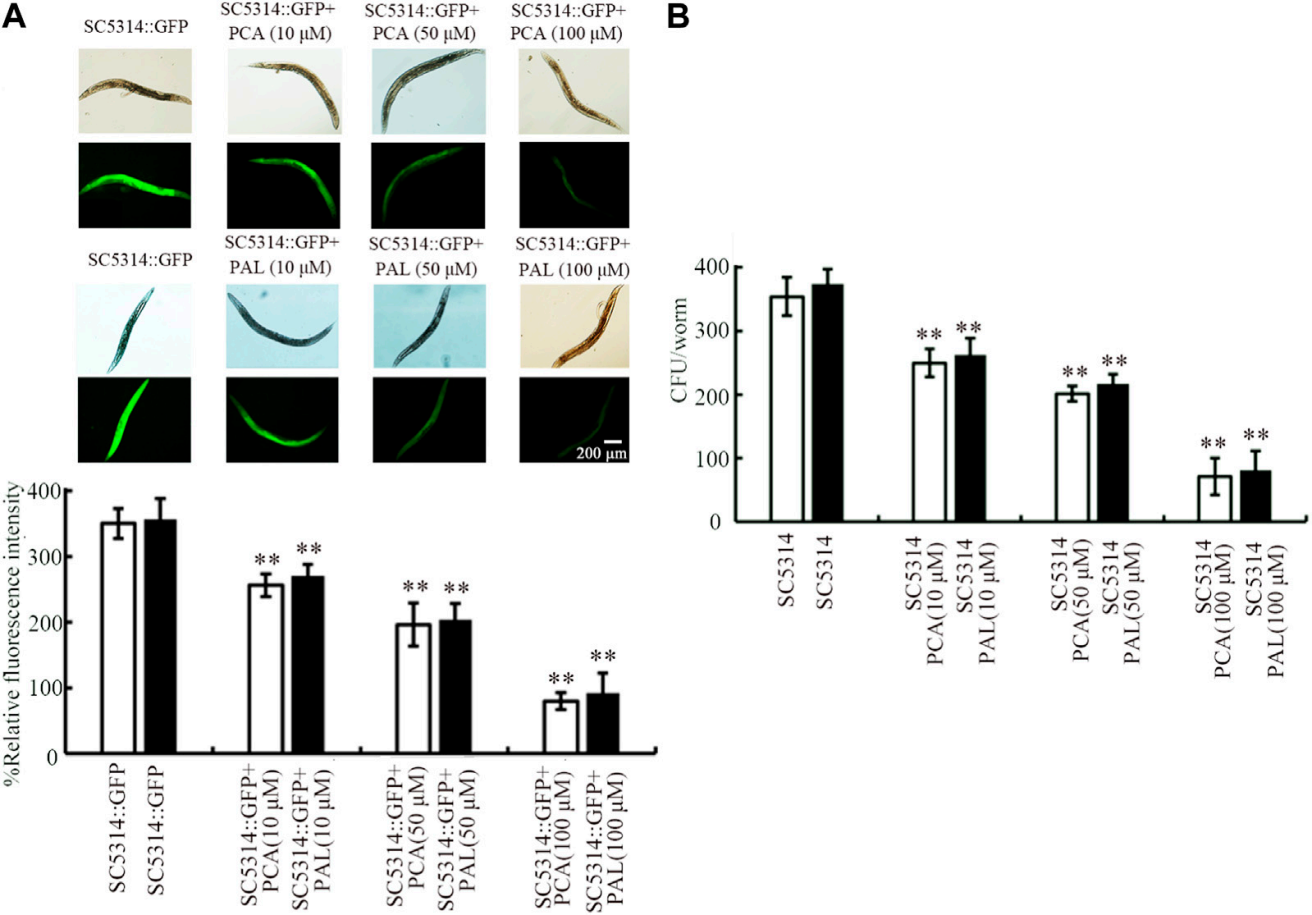
Effect of PCA and PAL treatment on *C. albicans* accumulation in intestinal lumen of nematodes. **(A)** Effect of PCA and PAL treatment on relative fluorescence intensity of SC5314:GFP in intestinal lumen of nematodes. **(B)** Effect of PCA and PAL treatment on CFU of SC5314 in infected nematodes. PCA, protocatechuic acid; PAL, protocatechuic aldehyde. ^**^ *p <* 0.01 vs*.* SC5314:GFP or SC5314.

To determine the underlying mechanism for observed benefits of PCA and PAL against fungal infection, SC5314 accumulation in intestinal lumen was investigated. After the infection, pronounced SC5314:GFP accumulation could be detected in intestinal lumen of nematodes (Figure 2A). The SC5314:GFP accumulation in intestinal lumen could be inhibited by treatment with 10–100 μM PCA and 10–100 μM PAL (Figure 2A). After the infection, a high level of intestinal CFU of SC5314 was further detected (Figure 2B). Moreover, the intestinal CFU of SC5314 after infection could be significantly suppressed by treatment with 10–100 μM PCA and 10–100 μM PAL (Figure 2B). The inhibition in intestinal SC5314:GFP accumulation and intestinal CFU by PCA and PAL was concentration dependent, and the 100 μM PCA and 100 μM PAL showed the most beneficial effect against SC5314 accumulation in intestinal lumen (Figures 2A,B).

### Effect of PCA and PAL on innate immune response after fungal infection

**FIGURE 3:**
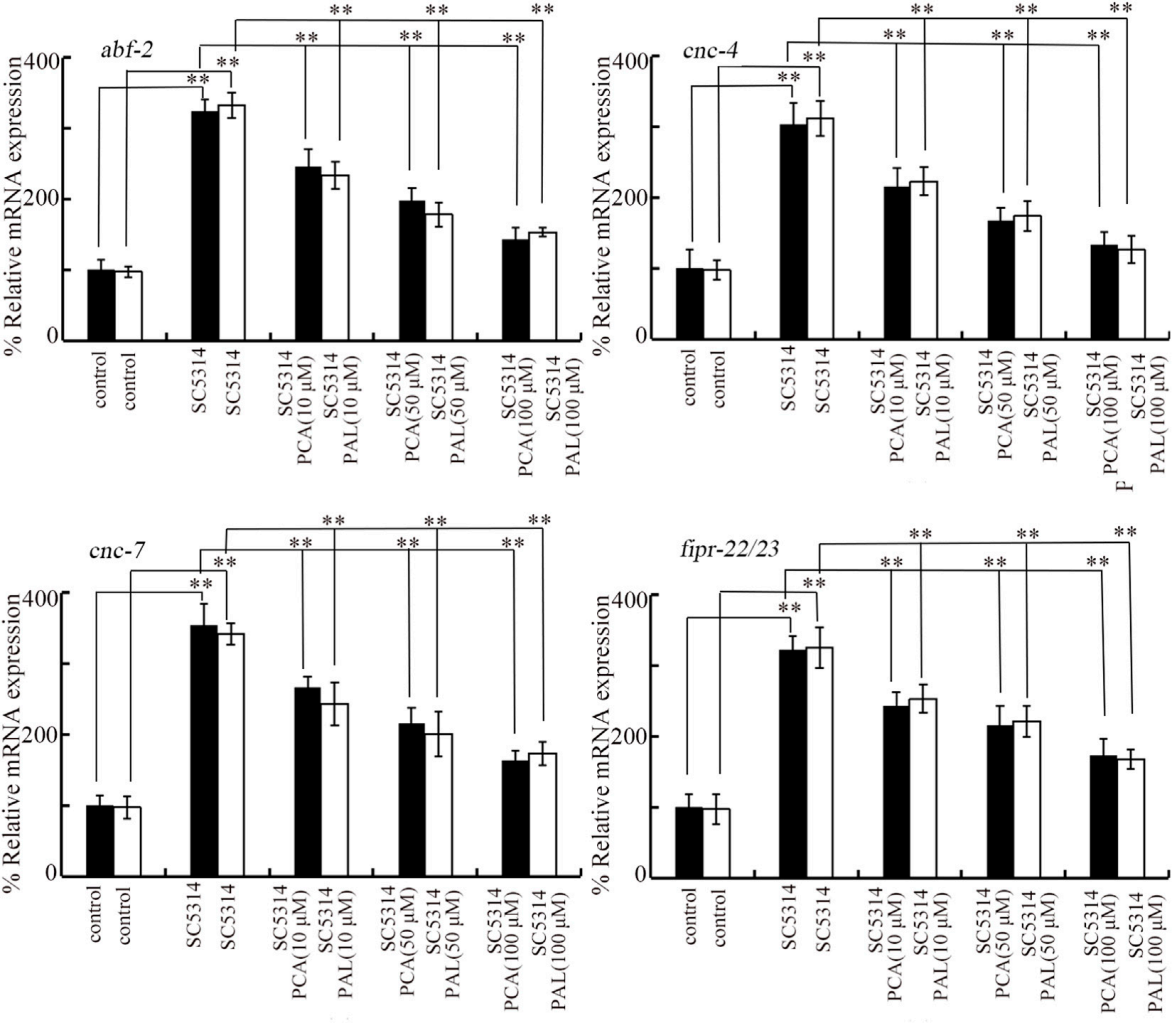
Effect of PCA and PAL treatment on expressions of *abf-2*, *cnc-4*, *cnc-7*, and *fipr-22/23* in *C. albicans* infected nematodes. PCA, protocatechuic acid; PAL, protocatechuic aldehyde. ^**^ *p <* 0.01.

Four genes (*abf-2*, *cnc-4*, *cnc-7*, and *fipr-22/23*) were used as antimicrobial genes in response to SC5314 infection (Sun et al., 2016a). The noticeable increase in expression of these 4 antimicrobial genes was induced by SC5314 infection (Figure 3). After SC5314 infection, the increase in expression of these 4 antimicrobial genes was obviously suppressed by treatment with 10–100 μM PCA (Figure 3). Similarly, the increase in expression of these 4 antimicrobial genes in SC5314 infected nematodes was also significantly inhibited by 10–100 μM PAL (Figure 3). The effect of PCA and PAL to modulate expressions of *abf-2*, *cnc-4*, *cnc-7*, and *fipr-22/23* in SC5314 infected nematode was also concentration dependent (Figure 3).

### Effect of PCA and PAL against *C. albicans* infection depended on p38 MAPK signaling and insulin signaling.

**FIGURE 4:**
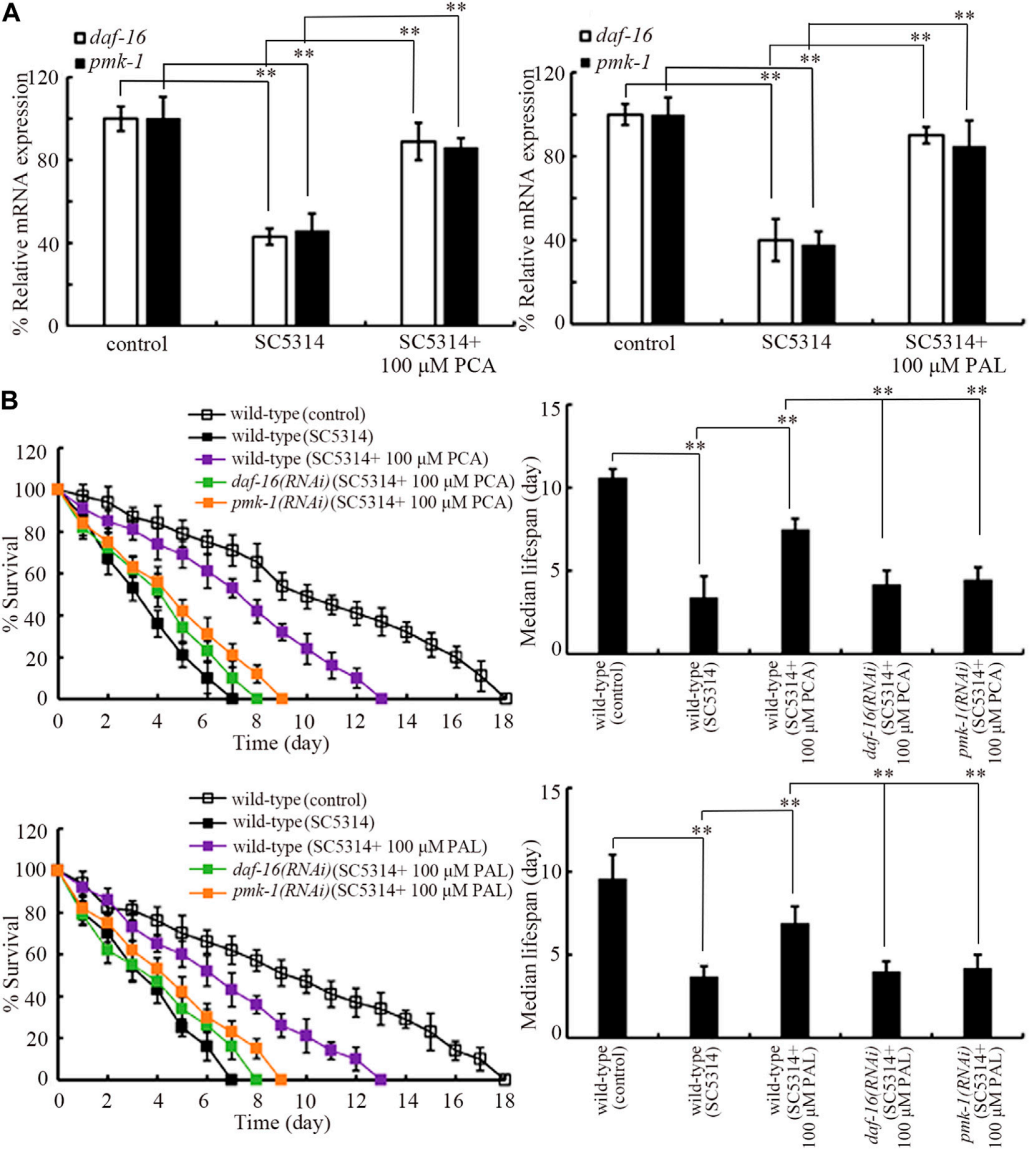
Effect *pmk-1* or *daf-16* RNAi on function of PCA and PAL against *C. albicans* infection. **(A)** Effect of PCA and PAL treatment on expressions of *pmk-1* and *daf-16* in *C. albicans* infected nematodes. **(B)** Effect *pmk-1* or *daf-16* RNAi on function of PCA and PAL in increasing survival of *C. albicans* infected nematodes. RNAi of *pmk-1* or *daf-16* was performed after *C. albicans* infection. PCA, protocatechuic acid; PAL, protocatechuic aldehyde. ^**^ *p <* 0.01. Lifespan curves of SC4314 showed a significant difference (*p* < 0.01) compared to control. Lifespan curves of SC5314 + 100 μM PCA and SC5314 + 100 μM PAL showed a significant difference (*p* < 0.01) compared to SC5314. Lifespan curves of *daf-16(RNAi)* (SC5314 + 100 μM PCA) and *pmk-1(RNAi)* (SC5314 + 100 μM PCA) showed a significant difference (*p* < 0.01) compared to the group of SC5314 + 100 μM PCA. Lifespan curves of *daf-16(RNAi)* (SC5314 + 100 μM PAL) and *pmk-1(RNAi)* (SC5314 + 100 μM PAL) showed a significant difference (*p* < 0.01) compared to the group of SC5314 + 100 μM PAL.

Insulin and p38 MAPK are two normally determined signaling pathways required for controlling innate immunity (Millet and Ewbank, 2004). In p38 MAPK signaling pathway, PMK-1 is p38 MAPK. In insulin signaling pathway, DAF-16 is FOXO transcriptional factor. SC5314 infection only could cause the decrease in expressions of *daf-16* and *pmk-1* (Figure 4A). In SC5314 infected animals, treatment with 100 μM PCA and 100 μM PAL increased expression of *daf-16* and *pmk-1* (Figure 4A).

Moreover, the effect of 100 μM PCA and 100 μM PAL in increasing survival of SC5314 infected nematodes was significantly inhibited by RNAi of *daf-16* and *pmk-1* (Figure 4B). In addition, *daf-16* and *pmk-1* RNAi also significantly suppressed the function of 100 μM PCA and 100 μM PAL in decreasing both intestinal SC5314:GFP accumulation and intestinal CFU (Supplementary Figures S2A, B). Therefore, both PMK-1 and DAF-16 were required for the effect of PCA and PAL against *C. albicans* infection.

### PCA and PAL did not exhibit obvious antifungal activity

**FIGURE 5:**
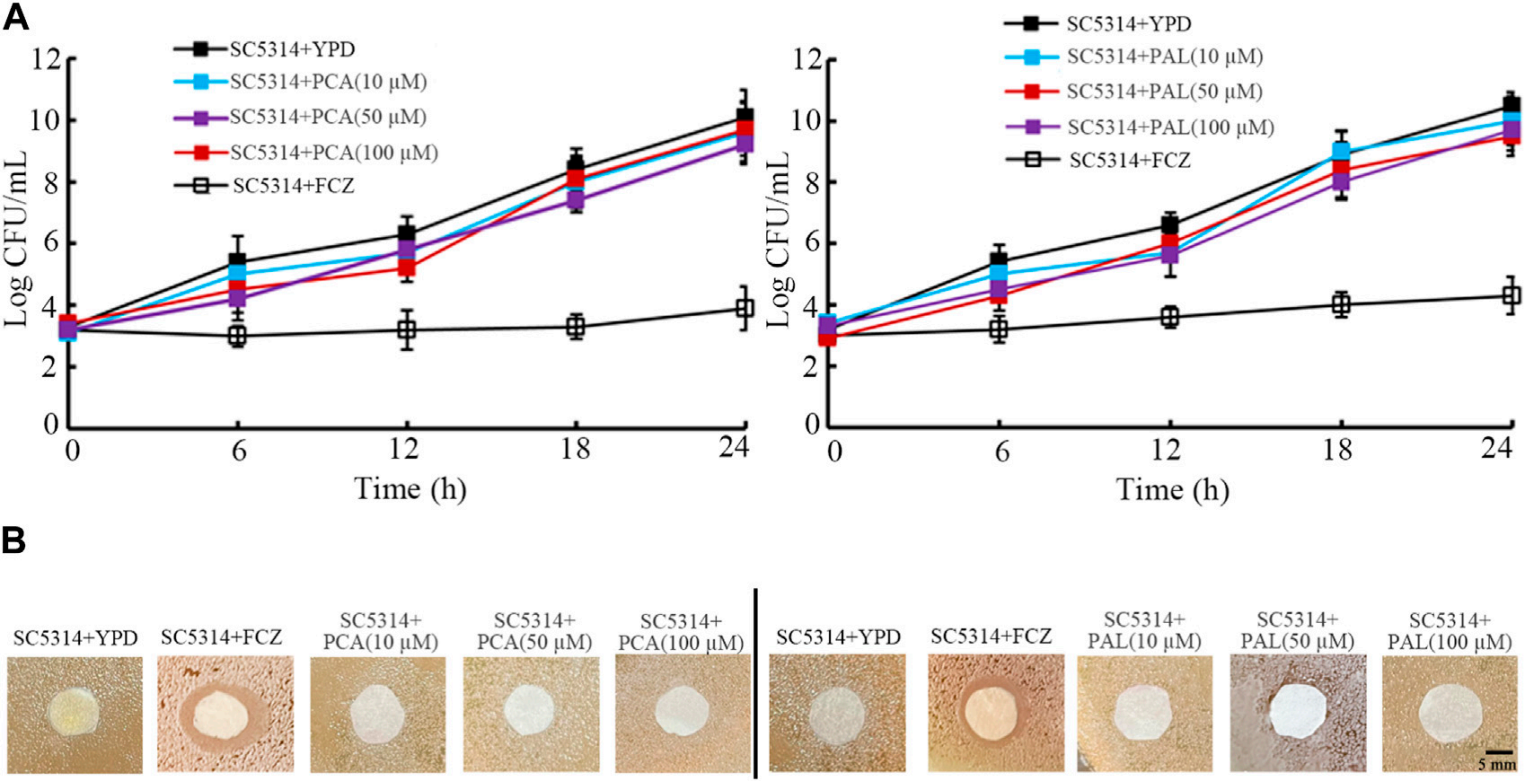
Analysis of antifungal activity of PCA and PAL. **(A)** Time-killing assay. **(B)** Disk diffusion assay. PCA, protocatechuic acid; PAL, protocatechuic aldehyde; FCZ, fluconazole. FCZ treatment concentration was 8 μg/mL.

Firstly, in time-kill assay, compared with strong anti-fungal activity of fluconazole (8 μg/mL), both 10–100 μM PCA and 10–100 μM PAL did not show noticeable anti-fungal activity from 6-h to 24-h (Figure 5A). Moreover, compared with obvious zone of inhibition induced by fluconazole (8 μg/mL), both 10–100 μM PCA and 10–100 μM PAL had no obvious effect on *C. albicans* SC5314 in the agar diffusion assay (Figure 5B).

### Effect of PCA and PAL on fungal biofilm formation

**FIGURE 6:**
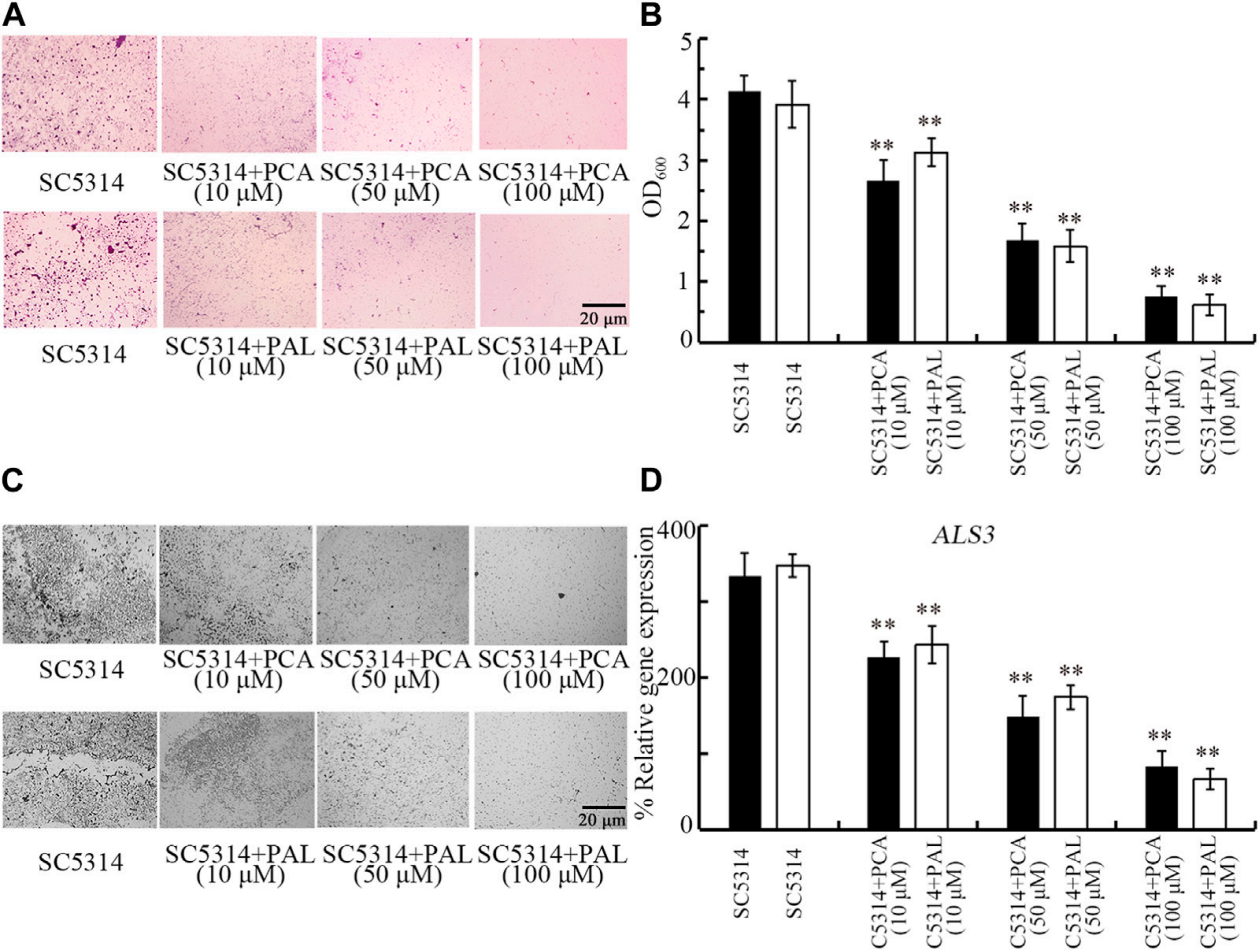
Effect of PCA and PAL on *C. albicans* biofilm formation. **(A)** Crystal violate staining images. **(B)** Effect of PCA and PAL on amount of biofilm formation based on OD_600_ absorbance analysis. **(C)** Effect of PCA and PAL on biofilm formation visualized under a light microscope. **(D)** Effect of PCA and PAL on expression of *ALS3*. PCA, protocatechuic acid; PAL, protocatechuic aldehyde. ^**^ *p <* 0.01 vs*.* SC5314.

Biofilm formation contributes to pathogenesis of *C. albicans* during their infection in hosts (Zeng et al., 2017). Both crystal violet staining and analysis of OD_600_ absorbance indicated that the biofilm formation of SC5314 was significantly reduced by 10–100 μM PCA and 10–100 μM PAL (Figures 6A,B). The beneficial effect of 10–100 μM PCA and 10–100 μM PAL in reducing biofilm formation of SC5314 was also found by visualization under light microscopy (Figure 6C). In addition, expression of biofilm-related gene *ALS3* was significantly decreased by treatment with both 10–100 μM PCA and 10–100 μM PAL (Figure 6D). Therefore, PCA and PAL treatment showed the inhibitory effect on biofilm formation in *C. albicans*.

### Effect of PCA and PAL on *C. albicans* hyphal growth

**FIGURE 7:**
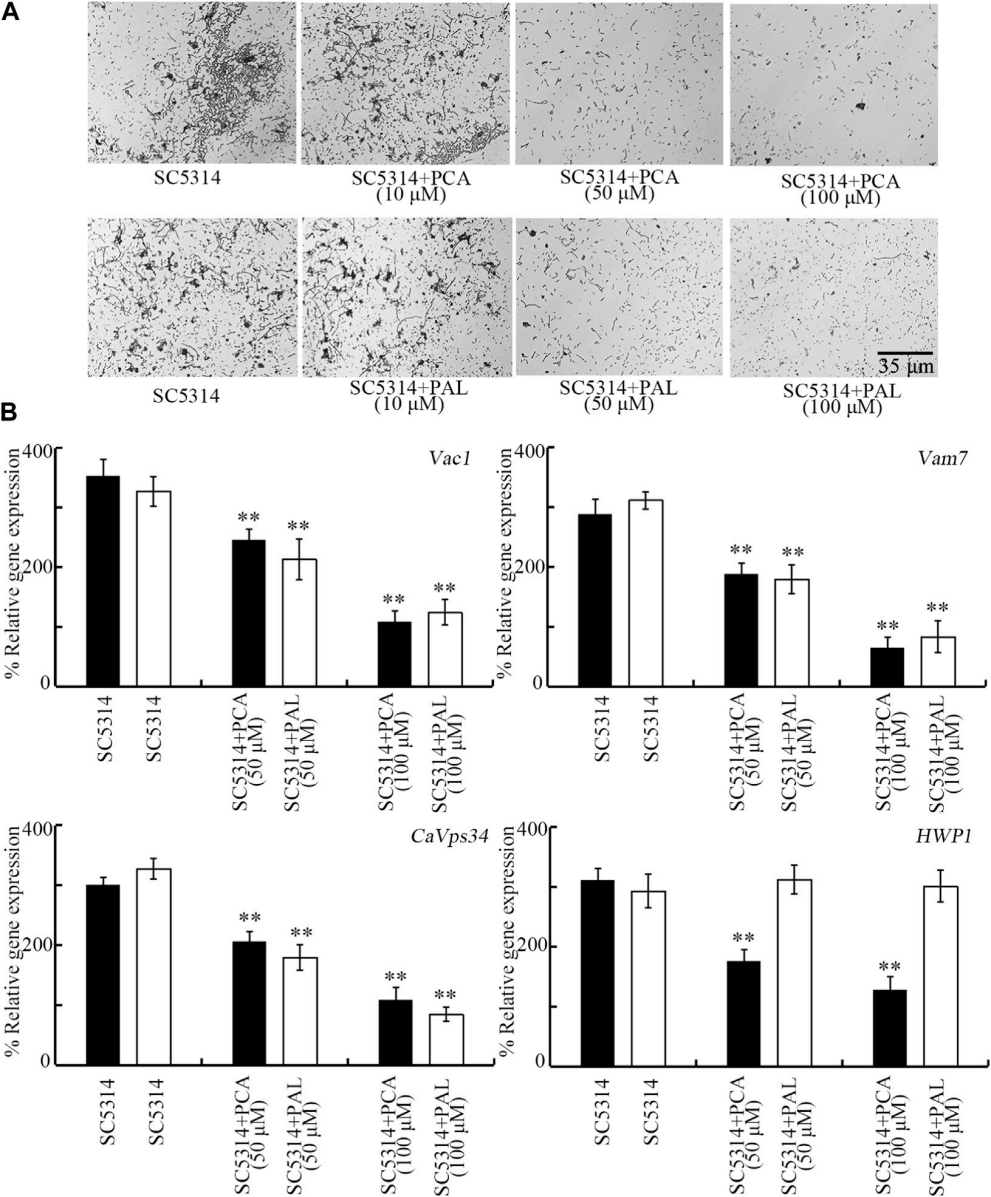
Effect of PCA and PAL on *C. albicans* hyphal growth. **(A)** Effect of PCA and PAL on hyphal growth. **(B)** Effect of PCA and PAL on expressions of *Vac1*, *Vma7*, *CaVps34*, and *HWP1*. PCA, protocatechuic acid; PAL, protocatechuic aldehyde. ^**^ *p <* 0.01 vs*.* SC5314.

Transition from yeast to hyphae also contributes to the induction of *C. albicans* pathogenicity (Gow et al., 2011). Although 10 μM PCA and PAL did not affect hyphal growth, the hyphal growth was obviously inhibited by 50 and 100 μM PCA and PAL (Figure 7A). After 50 and 100 μM PCA and PAL treatment, more yeast cells could be observed than the hyphal cells (Figure 7A).

During hyphal growth of *C. albicans*, *Vac1*, *Vam7*, *CaVps34*, and *HWP1* play important roles (Sharkey et al., 1999; Bruckmann et al., 2000; Poltermann et al., 2005; Franke et al., 2006). After treatment with 50 and 100 μM PCA, expressions of all these 4 genes were significantly decreased (Figure 7B). In addition, after treatment with 50 and 100 μM PAL, expressions of *Vac1*, *Vam7*, and *CaVps34* were also significantly decreased (Figure 7B). Therefore, both PCA and PAL treatment suppressed the transition from yeast to hyphae for *C. albicans* cells.

## Discussion

Till now, the reported pharmacological effects of PCA contain inhibition in neurodegenerative diseases, anti-oxidation, anti-inflammation, anti-hyperglycemia, and anti-aging (Khan et al., 2015; Semaming et al., 2015; Krzysztoforska et al., 2019). In addition, PAL treatment has been shown to have the pharmacological effects of neuroprotection, anti-oxidation, and inhibition in pulmonary fibrosis, sepsis, and diabetic nephropathy (Zhang et al., 2015; Chang et al., 2021; Zhang et al., 2021; Guo et al., 2022). We used *C. elegans* as the host to examine the possible effect of PCA and PAL against fungal infection. Due to high sensitivity to exposure, *C. elegans* is helpful for detecting pharmacological effects of compounds at different concentrations (Wang, 2020). Besides this, considering the well-described molecular background, *C. elegans* will provide an important platform to elucidate underlying mechanism for the observed pharmacological effects of certain compound (Bulteriis and Braeckman, 2020).

Previous reports have indicated that treatment with PCA was helpful for nematodes against heat stress, osmotic stress, and oxidative stress (Kim et al., 2014; Schmitt et al., 2021). In addition, treatment with PAL could delay paralysis and inhibit aggregation of Aβ plaques, suggesting its neuroprotective effect (Shi et al., 2023). We found that treatment with both 10–100 μM PCA and 10–100 μM PAL inhibited adverse effect of SC5314 infection in decreasing lifespan (Figure 1). Treatment with 10–100 μM PCA and 10–100 μM PAL could not alter lifespan of nematodes (Supplementary Figure S4), suggesting that this effect of PCA and PAL was not associated with the possible role of PCA and PAL in extending longevity. Our results here demonstrated novel therapeutic potential of PCA and PAL. That is, this suggests that PCA and PAL administration in the clinical may be helpful to reduce fatality rate caused by fungal pathogen infection to a certain degree. Besides this, PCA treatment has also been shown to have the function against virus infection in mice (Wang et al., 2022). Nevertheless, we also noted that treatment with PCA and PAL at the examined concentrations did not recover lifespan of SC5314 infected nematodes to control level (Figure 1).

After fungal infection, the colony formation in intestine normally acts as a crucial contributor to toxicity of pathogen infection (Pukkila-Worley et al., 2011). The identified important cellular contributor to antifungal infection function of PCA and PAL was the inhibition in SC5314 accumulation in intestinal lumen (Figure 2). This suggested that treatment with PCA and PAL may be helpful for the excretion of *C. albicans* from intestinal lumen in nematodes. The *C. albicans* accumulation could also be decreased by 64 mg/L thymol (Shu et al., 2016). Similarly, the beneficial effect of treatment with paeoniflorin or Xuebijing (a traditional Chinese medicine) in enhancing excretion of bacterial pathogen from intestine was also observed in nematodes (Zhang et al., 2022; Wang et al., 2023). Different from these, multi-walled carbon nanotubes enhanced toxicity of fungal infection by increasing accumulation of SC5314 in intestinal lumen (Shakoor et al., 2016).

Certain antimicrobial genes of nematodes will be activated by *C. albicans* infection to be against the adverse effects of fungal pathogen and kill the *C. albicans* cells (Pukkila-Worley et al., 2011; Sun et al., 2016a). We observed that the increase in expressions of antimicrobial genes (*fipr-22/23*, *cnc-7*, *cnc-4*, and *abf-2*) in SC5314 infected nematodes was suppressed by the following treatment with 10–100 μM PCA and PAL (Figure 3). The inhibition in colony formation and accumulation of SC5314 in intestine induced by PCA or PAL treatment may lead to this suppression in increase in expression of *fipr-22/23*, *cnc-7*, *cnc-4*, and *abf-2* in SC5314 infected animals. This further implies that inhibition in fungal pathogen accumulation in intestinal lumen may be the crucial cellular contributor to PCA and PAL function against fungal infection.

Moreover, RNAi of *pmk-1* or *daf-16* suppressed the formation of beneficial effect of PCA and PAL against fungal infection (Figure 4B). Therefore, the beneficial effect of PCA and PAL against fungal infection was dependent of p38 MAPK signaling and insulin signaling. p38 MAPK and insulin are conserved signaling pathways involved in controlling both innate immunity and stress responses (Harding and Ewbank, 2010; Zhao et al., 2016; Shao et al., 2019; Wang, 2019). Insulin signaling and p38 MAPK signaling are required for controlling innate immunity to both bacterial and functional infections (Troemel et al., 2006; Evans et al., 2008; Sun et al., 2016a; Kong et al., 2019). Mutation of *daf-16* or *pmk-1* caused the decrease in lifespan, enhancement in pathogen accumulation in intestinal lumen, and reduction in expression of antimicrobial genes (such as *abf-2*) in fungal infected nematodes (Sun et al., 2016a). Beneficial effect of thymol against fungal infection also required function of p38 MAPK signaling pathway (Shu et al., 2016). Meanwhile, in SC5314 infected nematodes, expression of *pmk-1* and *daf-16* could be increased by PCA and PAL treatment (Figure 4A). For the underlying molecular basis, our data suggests that PCA and PAL have the function against *C. albicans* infection by activating insulin signaling and p38 MAPK signaling in nematodes. It was also reported that PCA treatment could significantly upregulate expression of *daf-16* (Dilberger et al., 2019).

Besides the inhibition in fungal pathogen accumulation in intestinal lumen, antifungal activity is another possible mechanism for formation of anti-fungal infection property of bioactive compounds. Nevertheless, time-kill assay indicated that both 10–100 μM PCA and 10–100 μM PAL had no obvious anti-fungal effect (Figure 5A). In addition, we also did not observe obvious zone of inhibition after treatment with 10–100 μM PCA and PAL in the agar diffusion assay (Figure 5B). These observations indicated that anti-fungal infection property of PCA and PAL in nematodes may be not directly associated with possible effect of anti-fungal activity for PCA and PAL.

Biofilm formation potentially protects *C. albicans* from the defense of host immune system (Chandra and Mukherjee, 2015). More importantly, the formed *C. albicans* biofilm is very resistant to traditional antifungal agents by strongly attaching to biotic or abiotic surfaces (Oppenheimer-Shaanan et al., 2013). We further observed the obvious beneficial effect of PCA and PAL treatment in reducing SC5314 biofilm formation (Figures 6A–C). Our data suggested that administration with PCA and PAL will be helpful for enhancing antifungal agents during treatment for fungal infections in patients. It has been reported that treatment with PCA showed the inhibitory effects on biofilms formation of *E. coli* (Bernal-Mercado et al., 2018). In addition, treatment with PAL had the inhibitory effects on biofilms formation of *Ralstonia solanacearum*, *Yersinia enterocolitica* and *Vibrio parahaemolyticus* (Li et al., 2016; Liu and Wang, 2022; Meng et al., 2022).

*C. albicans* biofilm is a cellular community encased in self-released extracellular polysaccharides (Rodrigues et al., 2018). In *C. albicans*, *ALS3* encode a cell wall glycoprotein, and acts at adherence step of biofilms formation (Roudbarmohammadi et al., 2016). We found that the ALS3 expression was significantly decreased by 10–100 μM PCA and PAL treatment (Figure 6D), which provides an important molecular basis for PCA and PAL treatment in reducing fungal biofilm formation to a certain degree.

Moreover, we observed that the *C. albicans* hyphal growth was significantly inhibited by 50 and 100 μM PCA and PAL treatment (Figure 7A), which suggested the inhibition in transition from yeast to hyphal cells. After the *C. albicans* biofilm formation, the hyphae will appear together with the extracellular matrix material production (Qian et al., 2020). Hyphal growth is another important virulence factor, since the formed hyphae potentially attach to cells and cause damage on tissues in hosts (Finkel and Mitchell, 2011; Tati et al., 2016). Hyphal growth is closely associated with the biofilm formation, and hyphae are intertwined with biofilms in *C. albicans* (Finkel and Mitchell, 2011). Our data suggested that PCA and PAL treatment can provide a useful strategy to inhibit formation of both hyphae and biofilms produced by pathogenic C. albicans.

Furthermore, we found that expressions of some genes governing the *C. albicans* hyphal growth were downregulated by PCA and PAL treatment (Figure 7B), which further provides important molecular basis for PCA and PAL treatment in suppressing fungal hyphal growth. After 50 and 100 μM PCA and PAL treatment, the expressions of hyphae-related genes (*Vac1*, *Vma7*, *CaVps34*, and/or *HWP1*) were significantly decreased (Figure 7B). In *C. albicans*, null mutation of *Vac1* encoding vesicle transporter caused defective in hyphal growth (Franke et al., 2006). The Vam7, a H^+^-ATPase subunit, regulates vacuolar ion transport, which is required for normal hyphal growth (Poltermann et al., 2005). Null mutation of *CaVps34* encoding a phosphatidylinositol 3-kinase resulted in the significant delay in yeast-to-hyphae transition (Bruckmann et al., 2000). Deletion of *HWP1* encoding a hypha-specific cell surface protein caused deficit in hyphal development (Sharkey et al., 1999).

## Conclusion

In conclusion, treatment with PCA and PAL effectively inhibited adverse effect of fungal infection in decreasing lifespan of nematodes. This beneficial effect of PCA and PAL treatment was largely due to the suppression in *C. albicans* accumulation in intestinal lumen. Both p38 MAPK signaling and insulin signaling were required for formation of beneficial effect of PCA and PAL against fungal infection. Moreover, both biofilm formation and hyphal growth of *C. albicans* were inhibited by PCA and PAL treatment, suggesting their anti-virulence potential. Our data suggested the anti-fungal infection anti-virulence potentials of PCA and PAL treatment. Nevertheless, the underlying mechanism of anti-virulence potential for PCA and PAL needs to be further determined. The further identification of direct pharmacological targets for PCA and PAL against fungal virulence is suggested to be further performed.

## Data Availability

The original contributions presented in the study are included in the article/Supplementary Material, further inquiries can be directed to the corresponding author.
