# Supplementary material for: Procatechuic acid and protocatechuic aldehyde increase survival of Caenorhabditis elegans after fungal infection and inhibit fungal virulence
Source: Front Pharmacol. 2024 May 22;15:1396733. doi: 10.3389/fphar.2024.1396733 (PMC11150623; doi:10.3389/fphar.2024.1396733)
Supplement: Supplementary file 1 [file DataSheet1.doc]

**Supporting Information:**


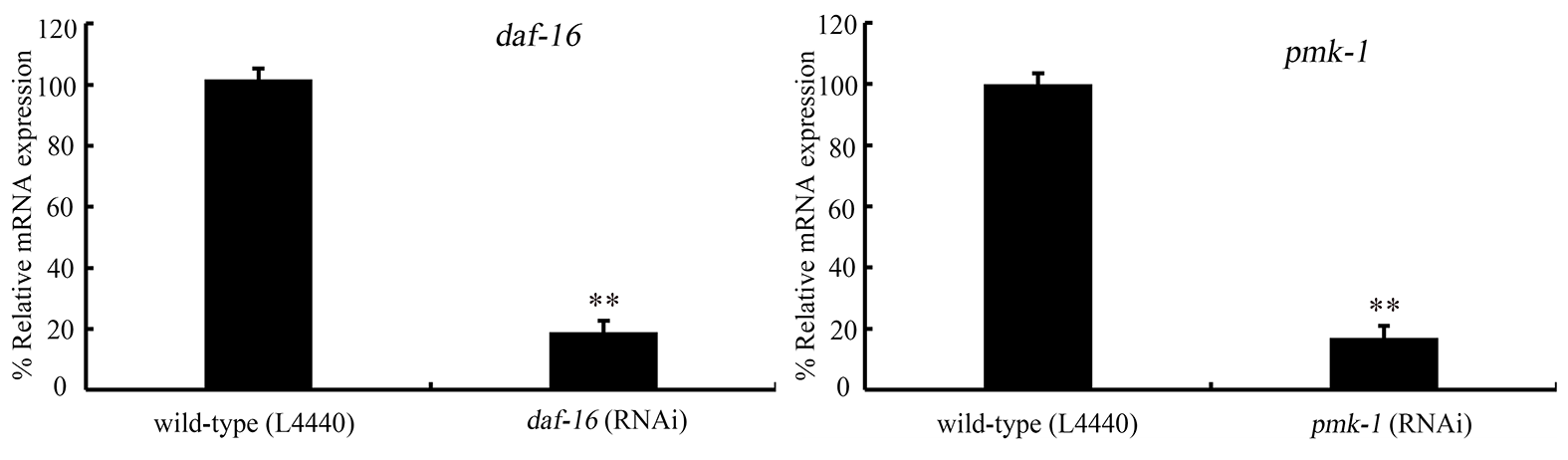


**Fig. S1** Efficiency of RNAi knockdown of *pmk-1* and *daf-16* in wild-type nematodes. ^**^*P <* 0.01 *vs* wild-type(L4440).


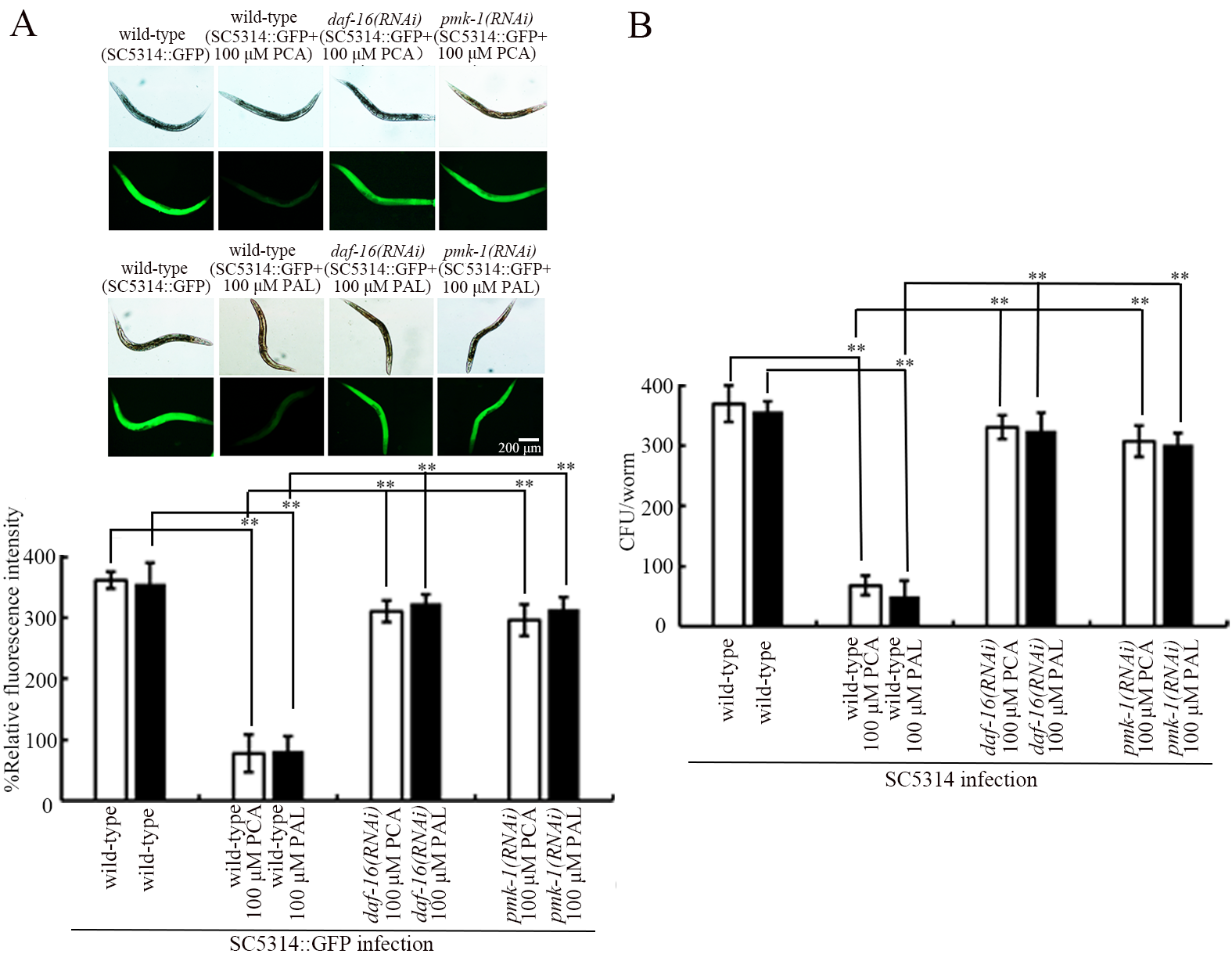


**Fig. S2** Effect *pmk-1* or *daf-16* RNAi on function of PCA and PAL in decreasing relative fluorescence intensity of SC5314::GFP in intestinal lumen (A) and in reducing CFU of SC5314 (B) in infected nematodes. RNAi of *pmk-1* or *daf-16* was performed after *C. albicans* infection. ^**^*P <* 0.01.


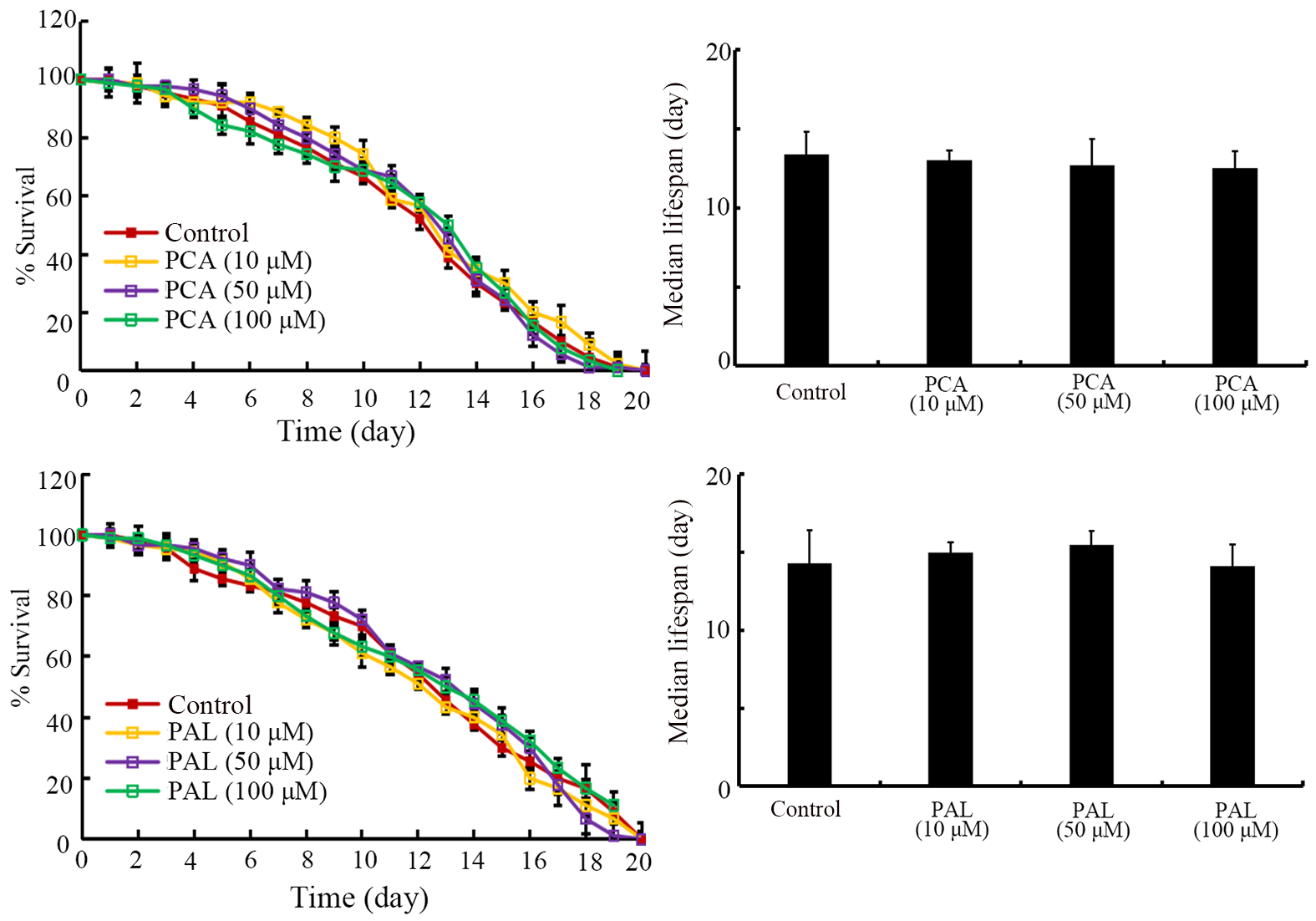


**Fig. S3** Effect of PCA and PAL treatment for adult stage for 24 h on lifespan of nematodes.

**Table S1.** Information for *C. albicans* strains

| Strain | Description |
| --- | --- |
| SC5314 | clinical isolate |
| CaSA1 | ura3::imm434/ura3::imm434; *CDR1-GFP-URA3* |

Note: In *C. albicans*, the *CDR1* gene encodes an ABC transporter that functions as an efflux pump, which is involved in the control of pathogenic adaptation.

**Table S2.** Primer information for qRT-PCR in *C. elegans*

| Gene | Forward primer (5’-3’) | Reverse primer (5’-3’) |
| --- | --- | --- |
| *abf-2* | TGGTAATGCACAACCCCTGA | TTCGTCCGTTCCCTTTTCCT |
| *cnc-4* | GCTTCGCTACATTCTCGTCCT | GTATCCACCACCATACCCGC |
| *cnc-7* | GGACGGTACATTCCCATACC | CAGGTTCAATGCAGTATGGCTATGG |
| *fipr-22/23* | GCTGAAGCTCCACACATCC | TATCCCATTCCTCCGTATCC |
| *daf-16* | ACCGTTGGTCAAATGCTTGC | TGGCTTCTTACGACAACGCT |
| *pmk-1* | TCCGACTCCACGAGAAGGAT | CACGATATGTACGACGGGCA |
| *tba-1* | TCAACACTGCCATCGCCGCC | TCCAAGCGAGACCAGGCTTCAG |

**Table S3.** Primer information for qRT-PCR in *C. albicans* SC5314

| Gene | Forward primer (5’-3’) | Reverse primer (5’-3’) |
| --- | --- | --- |
| *CaVps34* | CAACCACAAAGCGCACTACC | CCGCACCGGTGTACTGATTA |
| *Vma7* | TCTCCAACGAACCAGGCAAA | ACGGATGATCTTTGGATGGGA |
| *Vac1* | CTCGTAGGCGACAATGACGA | CTTAGTAAGCCGCGGAGGAG |
| *HWP1* | TCTACTGCTCCAGCCACTGA | CCAGCAGGAATTGTTTCCAT |
| *ALS3* | AATGCTGTTTTGGGTTGGTC | TCACCTGCCTGAAATTGACA |
| *18S rRNA* | CGATGGAAGTTTGAGGCAATA | CTCTCGGCCAAGGCTTATACT |
